# Efficient network immunization under limited knowledge

**DOI:** 10.1093/nsr/nwaa229

**Published:** 2020-09-03

**Authors:** Yangyang Liu, Hillel Sanhedrai, GaoGao Dong, Louis M Shekhtman, Fan Wang, Sergey V Buldyrev, Shlomo Havlin

**Affiliations:** Department of Systems Science, National University of Defense Technology, Changsha, 410073, China; Department of Physics, Bar-Ilan University, Ramat Gan 5290002, Israel; Faculty of Science, Jiangsu University, Zhenjiang, 212013, China; Department of Physics, Bar-Ilan University, Ramat Gan 5290002, Israel; Networks Science Institute, Northeastern University, Boston, MA 02115; Department of Physics, Bar-Ilan University, Ramat Gan 5290002, Israel; Department of Physics, Yeshiva University, New York, New York 10033, USA; Department of Physics, Bar-Ilan University, Ramat Gan 5290002, Israel

**Keywords:** percolation, complex networks, network immunization, critical phenomena

## Abstract

Targeted immunization of centralized nodes in large-scale networks has attracted significant attention. However, in real-world scenarios, knowledge and observations of the network may be limited thereby precluding a full assessment of the optimal nodes to immunize (or quarantine) in order to avoid epidemic spreading such as that of the current COVID-19 epidemic. Here, we study a novel immunization strategy where only *n* nodes are observed at a time and the most central among these *n* nodes is immunized. This process can globally immunize a network. We find that even for small *n* (≈10) there is significant improvement in the immunization (quarantine) which is very close to the levels of an immunization with full knowledge. We develop an analytical framework for our method and determine the critical percolation threshold *p_c_* and the size of the giant component *P*_∞_ for networks with arbitrary degree distributions *P*(*k*). In the limit of *n* → ∞ we recover prior work on targeted immunization, whereas for *n* = 1 we recover the known case of random immunization. Between these two extremes, we observe that as *n* increases, *p_c_* increases quickly towards its optimal value under targeted immunization with complete information. In particular, we find a new general scaling relationship between |*p_c_*(∞) − *p_c_*(*n*)| and *n* as |*p_c_*(∞) − *p_c_*(*n*)| ∼ *n*^−1^exp ( − α*n*). For Scale-free (SF) networks, where *P*(*k*) ∼ *k*^−γ^, 2 < γ < 3, we find that *p_c_* has a transition from zero to non-zero when *n* increases from *n* = 1 to *O*(log *N*) (where *N* is the size of network). Thus, for SF networks, having knowledge of ≈log *N* nodes and immunizing them can dramatically reduce epidemic spreading. We also demonstrate our limited knowledge immunization strategy on several real-world networks and confirm that in these real networks, *p_c_* increases significantly even for small *n*.

## Teaser

A novel network immunization strategy is proposed and studied to provide a theoretical framework for understanding and carrying out efficient immunization with limited knowledge.

ORIGINAL UNEDITED MANUSCRIPT

* Equally contributed to this work
† Corresponding authors. E-mails: dfocus.gao@gmail.com; lsheks@gmail.com; havlins@gmail.com

## INTRODUCTION

Networks play a crucial role in many diverse systems [1–11]. Connectivity of components is critical for maintaining the functioning of infrastructures like the internet [12] and transportation networks [13], as well as for developing efficient immunization against epidemics [14] and the spread of information in social systems [15]. Due to this importance, researchers have long focused on how a network can be optimally immunized or fragmented to prevent epidemics or maintain infrastructure resilience [16–20]. Many approaches have used percolation theory from statistical physics to prevent the spread of viruses or assess network resilience under the infection or failure of some fraction of nodes or links [21–32].

Early studies in networks found that immunizing real networks against an epidemic is highly challenging due to the existence of hubs which prevent eradication of the virus even if many nodes are immunized [33–35]. At the same time, if these hub nodes, largest degree nodes, are targeted, the network can quickly be separated leading to immunity or failure [36]. However, these previous models of targeted immunization have assumed full knowledge of the network structure which in most cases is not available. Research has shown that even those in control or attack of a network often have knowledge of only a small portion of the whole network structure [37–41]. This has been further demonstrated with the current COVID-19 epidemic where the detailed social network of individuals is unknown.

In this paper, we study targeted immunization in networks with limited knowledge. Note that the methodology equally applies to efficient attacks (where the targeted nodes are removed) on networks. We assume that at each stage, a number $n$ nodes are observed and the node with highest degree is immunized and thus unable to continue spreading infection. This procedure is repeated until $1-p$ fraction of nodes are immunized. In particular, our model could apply to a situation where several cooperative teams are sent to immunize a network and each team has access to information on a small subset ($n$ nodes) of the network. We develop a theoretical framework for this model of immunization with limited information using percolation theory for networks with arbitrary degree distribution. In the limit of $n = N$ we recover prior work on targeted immunization [36], whereas for $n = 1$ we recover the case of random immunization [34, 35]. We observe excellent agreement between our theoretical framework and simulation results regarding the efficient of the immunization as measured by both the critical threshold $p_c$ and the size of the giant component $P_\infty$ for $p > p_c$ as a function of $n$. We note that a smaller giant component, implies improved immunization, whereas a larger critical threshold, $p_c$, is also more efficient since fewer quarantines/vaccines are needed to stop the epidemic. We find a scaling relationship between $n$ and $p_c$ for both Erdős-Rényi (ER) and Scale-Free (SF) networks, which we determine both analytically and through simulations. Surprisingly, we find that $p_c$ quickly reaches a plateau even for relatively small $n$ (of order 10), after which increasing $n$ has negligible effect on $p_c$. This suggest that obtaining information on even a small number ($n$) of people can lead to significant mitigation of the epidemic.

## RESULTS

### Model

Let $G(V, E)$ be a network where $V$ and $E$ are the set of nodes and edges, respectively. $N = |V|$ is the number of nodes in the network. We assume that a preventer has limited knowledge of the overall network structure and instead possesses only limited information on several nodes. Specifically, we randomly select $n$ nodes for which the immunizing team is assumed to obtain information on their node degree. We then immunize the node with the highest degree among these $n$. This is performed by many teams such that they collectively immunize a fraction of $1 - p$ nodes. The process lead to identical results if instead of immunizing the nodes they are put into quarantine. We note that the immunizing team only retains knowledge of the initial node degree and does not reduce the degree based on any neighbors that might have been immunized or quarantined. This is due to the fact that when asking individuals about their neighbors, the individuals are unlikely to know that a neighbor has recently been immunized or quarantined.

In Fig. 1, the limited information immunization is illustrated together with global targeted immunization on a network. Here a total of $n = 3$ nodes are observed. In panel (a), an individual with global information about the network structure chooses the highest degree node $u$ to immunize. However, in panel (b), the individual only knows at a time the degree of 3 nodes in the network, i.e. $v_1, v_2, v_3$. Consequently, node $v_3$ with the highest degree $k = 4$ (marked in red) is immunized.

ORIGINAL UNEDITED MANUSCRIPT

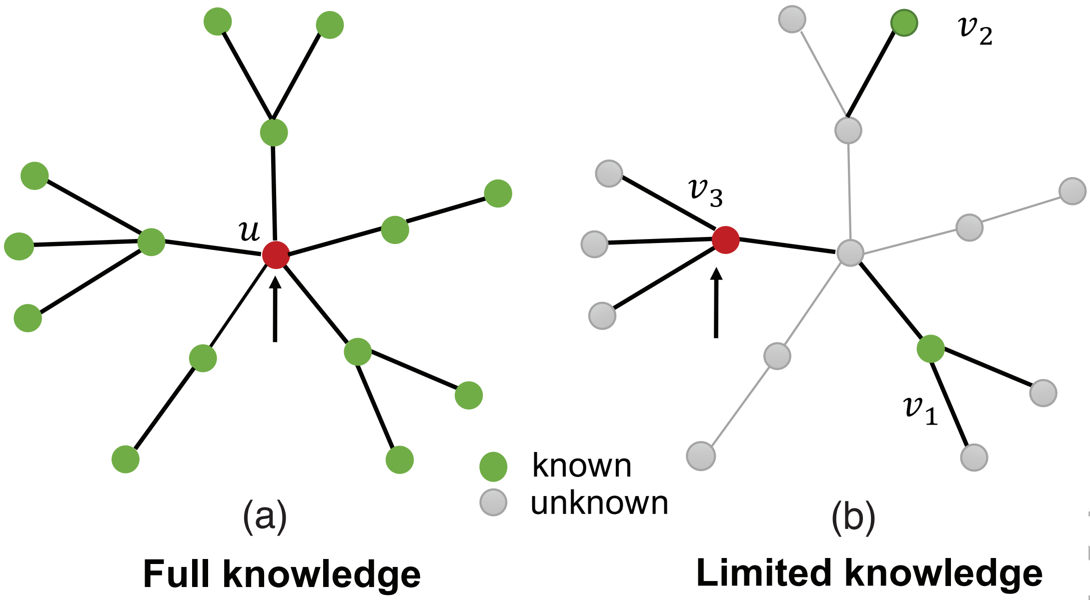

FIG. 1. Schematic illustration of our limited knowledge immunization strategy. The preventer is able to observe the degree of nodes that are colored green, while the gray nodes are unknown. (a) For the classical targeted immunization, one has complete information on the global structure of the network and chooses the highest degree node ($u$) to immunize. (b) Here the case of an individual with limited knowledge of the network is demonstrated. In this figure, we set $n = 3$ and only degrees of nodes $v_1$, $v_2$ and $v_3$ are known. Given this limited information, the preventer would choose to immunize $v_3$, being unaware that an unobserved higher degree node exists. At the next immunization, only nodes which have not been immunized yet will be observed. For another model that yields similar results see Fig. S4.

ORIGINAL UNEDITED MANUSCRIPT

### ER Networks

We now study our limited knowledge immunization strategy, i.e., the general result, Eqs. (12) and (13), on ER networks. First, we analyze the giant component $P_\infty$. For the case $n = 1$, limited knowledge immunization reduces to the classical random immunization, while for $n \to \infty$ (meaning the global network is observed) corresponds to targeted immunization [17, 33, 35, 36]. Using Eq. (13), the giant component $P_\infty$ can be solved numerically for any given $p$. In Fig. 2(a), simulations and analytic results are shown for the giant component $P_\infty$ as a function of $1 - p$ under limited information immunization with different $n$. As the knowledge index $n$ increases from 1 to $N$, limited knowledge immunization moves from being like random immunization to being like targeted immunization. The simulations are in excellent agreement with the theoretical results (lines).

Next, we focus on the critical threshold, $p_c$, of limited knowledge immunization. Overall, we find that one does not need a very large knowledge $n \sim 10$ to achieve nearly the very close effect as targeted immunization with complete information. This can be seen by observing the critical threshold $p_c$ as a function of $n$ in Fig. 2(b). In Fig. 2(c) we show the variation of $p_c$ with $\langle k \rangle$ for several fixed $n$.

Next, the behavior of $p_c$ in the limit of large $n$ is derived analytically. By examining Eq. (9) we notice that when $n \to \infty$ there are two distinct behaviors depending on whether $k$ is small, $F(k) < p$; or $k$ is large, $F(k) > p$. It can be shown (see Sec II.C in SM) that the leading term behaves as,

$$F_p(k) \sim \begin{cases} \frac{F(k)}{p} - \frac{1}{n}e^{-\alpha_k n}, & F(k) < p \\ 1 - \frac{1}{n}e^{-\alpha_k n}, & p < F(k) < 1 \\ 1, & F(k) = 1 \end{cases} \tag{1}$$

where $\alpha_k = |\log[p/F(k)]|$. In the limit $n \to \infty$, we can get the expected result for targeted immunization, $F_p(k) = \min\{F(k)/p,\ 1\}$ [35, 36].

Plugging Eq. (1) into Eq. (14), and noting that from a sum of exponentials decaying with $n$ only the lowest decay rate contributes to the leading term, we obtain (see Sec II.C in SM)

$$p_c(n) \sim p_c^\infty - A\frac{1}{n}e^{-\alpha n}, \tag{2}$$

where $p_c^\infty = p_c(n \to \infty)$, and the decay rate $\alpha$ is now

$$\alpha = \min_k \left|\log\left(p_c^\infty / F(k)\right)\right| .$$

The pre-factor $A = (2p_c^\infty k_{\text{slow}})/(k_> k_<)$, where $k_<$ is the largest degree such that $F(k) < p_c^\infty$, $k_> = k_< + 1$ and $k_{\text{slow}}$ is the degree which gives the lowest rate $\alpha$. (See illustration in SM).

It is clear that $k_{\text{slow}}$ must be $k_<$ or $k_>$ because $F(k)$ is monotonic. If $F(k_{\text{slow}}) = F(k_>) = 1$ then $k_<$ should be taken as $k_{\text{slow}}$, and the corresponding $\alpha$ should be taken. It should also be noted that if $k_{\text{slow}}$ is not unique, it would simply change the pre-factor $A$ in Eq. (2). Another special case is where $F(k_{\text{slow}}) = p_c^\infty$, then $|p_c^\infty - p_c| \sim 1/n$ (see Sec III in SM).

Fig. 2(d) shows $\Delta p_c = |p_c^\infty - p_c|$ as a function of $n$. As expected from the theory, one can see that $\Delta p_c \sim 1/n$ for small $n$ and exponential decay for large $n$. When $p_c \to 1$ which occurs for ER network when $\langle k \rangle \to 1$, the power law regime becomes much broader as explained in the Sec II.C of SM.

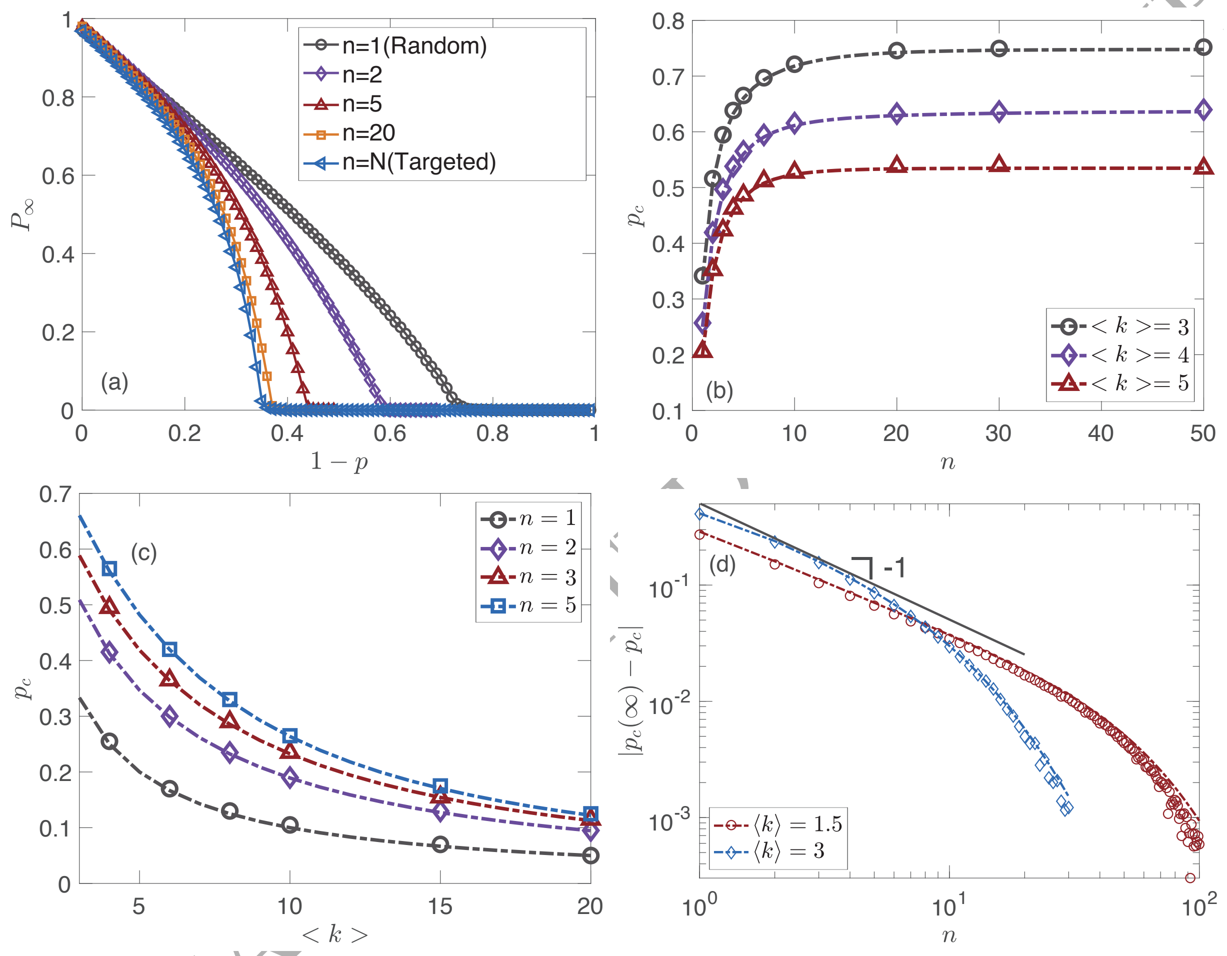

FIG. 2. Results on ER networks. (a) The giant component $P_\infty$ of an ER network with $\langle k \rangle = 4$ varies with the fraction of immunized nodes $1-p$ under limited knowledge. As the $n$ is increased, limited knowledge immunization tends to have the same immunization effect as targeted immunization. (b) The critical threshold $p_c$ of limited knowledge immunization as a function of $n$ on ER networks. The values of $p_c$ were determined from the $p$ of the maximal second largest clusters [28]. Note that already for small $n \sim 10$, $p_c$ is close to targeted immunization (global knowledge, $n \sim N$). (c) Critical threshold $p_c$ as a function the mean degree $\langle k \rangle$ of ER networks for limited knowledge immunization. (d) The scaling of $|p_c(\infty) - p_c|$ with $n$ on ER networks. Symbols are average results of simulations over 100 independent realizations on ER networks with $10^6$ nodes. All simulation results (symbols) agree well with theoretical results from Eq. (14) (dashed lines).

**SF Networks**

Next, we study SF networks with $P(k) = Ak^{-\gamma}, k = m, \cdots, K$, where $A = (\gamma - 1)m^{\gamma-1}$ is the normalization factor, and $m$ and $K$ are the minimum and maximum degree respectively [34]. Similar to ER networks, the size of the giant component, $P_\infty$ can be obtained from Eq. (13). Fig. 3(a), shows $P_\infty$ as a function of $1-p$ for different $n$ values. The results demonstrate that SF networks become more immunized compared to ER networks under the immunization as $n$ increases. Compared with ER networks, one can observe that slightly higher values of $n$ (more knowledge) are needed to reach the near-steady-state region of fully targeted strategy. Fig. 3(c) shows how $p_c$ varies with $\gamma$ for small $n$ for SF network where the minimum and maximum degree of network are $m = 2$ and $K = 1000$ respectively. Fig. 3 (d) shows $\Delta p_c = |p_c^\infty - p_c|$ as a function of $n$. As expected from the theory Eq. (12), the loglog plot captures the slope -1 for small $n$ while an exponential regime exists for large $n$.

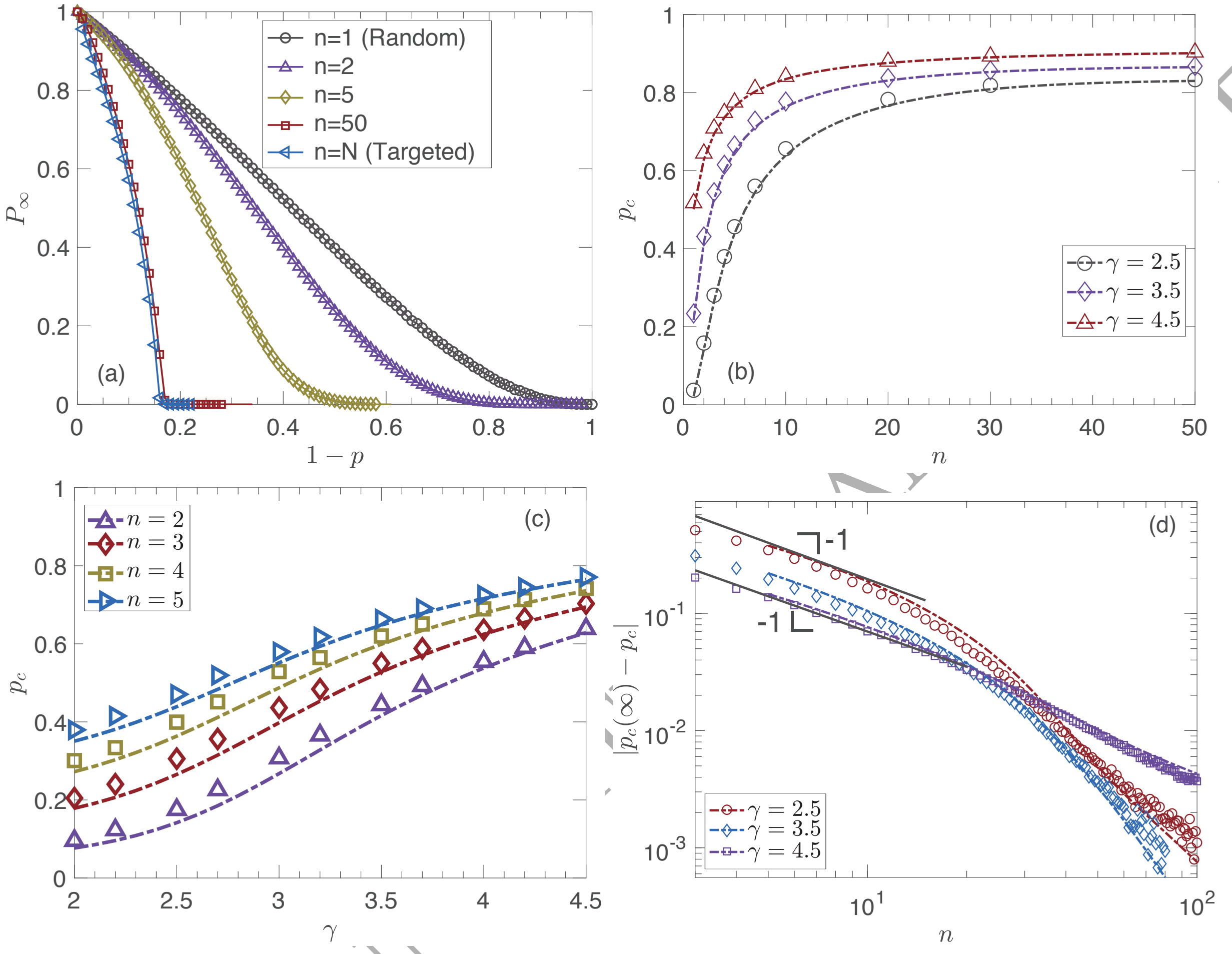

FIG. 3. Results for SF networks. Comparison of theory (lines) and simulation (symbols) for limited knowledge immunization, $n$, for SF networks. (a) The size of the giant component versus $n$ for SF network with $\gamma = 2.5$. (b) Critical threshold $p_c$ versus $n$ on SF networks. (c) The critical threshold, $p_c$, as a function of $\gamma$ for different values of $n$. (d) The log-log plot of $|p_c(\infty) - p_c|$ with $n$ on SF networks showing both a power law and exponential behavior. Simulations are obtained for networks of $10^6$ nodes and averages are taken over 100 independent realizations. The minimum and maximum degree of network are $m = 2$ and $K = 1000$ respectively. The theoretical results (dashed lines) are calculated from Eq. (14) are in excellent agreement with simulations (symbols).

For SF networks with $2 < \gamma < 3$, under random immunization ($n = 1$), it has been shown that $p_c = 0$ for an infinite system [34], while for high-degree immunization ($n \to \infty$), $p_c > 0$ [35, 36]. Next we wish to find out for which $n$, $p_c$ becomes non-zero, and how it depends on the system size $N$. To this end, we analyze Eqs. (9) and (14) for large $k$ (high degrees govern the behavior in SF), small $n$ and $p$ as follows (elaborated in Sec II.D of SM). It can be shown that for large degrees,

$$F(k) \approx 1 - (k/m)^{1-\gamma}.$$

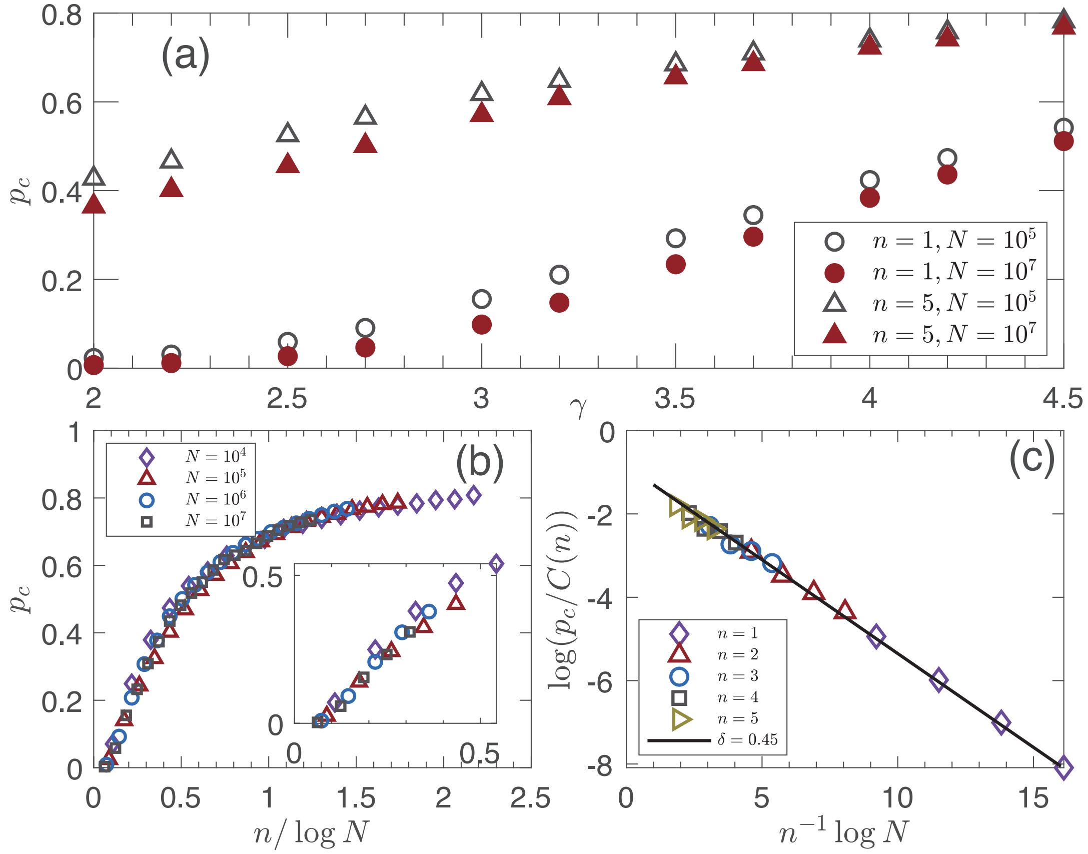

FIG. 4. How $p_c$ for SF networks depends on $\gamma$ and the system size $N$. (a) $p_c$ as a function of $\gamma$ for different values of $n$ and $N$. $p_c$ decreases with increasing $N$. For $n = 1$ and $2 < \gamma < 3$, it is well-known that $p_c$ approaches zero for infinite system. (b) $p_c$ as a function of $n/\log N$ for SF network with $\gamma = 2.1$. The enlarged figure for small $n = 1, \cdots, 5$ are shown in the inset, and $p_c$ approaches zero for $n/\log N \ll 1$. (c) The scaling of $p_c$ with $N$ and $n$ for large $N$ and small $n$. Here $C(n)$ is the pre-factor. The minimum and maximum degree of nodes are $m = 2$ and $K = N^{1/(\gamma-1)}$ respectively. This confirms Eq. (4) for $\gamma = 2.1$, and $\delta = (3-\gamma)/2 = 0.45$.

Substituting this into Eq. (9) and assuming $(k/m)^{\gamma-1} \gg n$ for large degrees, it can be concluded that

$$P_p(k) \approx \frac{\partial F_p(k)}{\partial k} \approx p^{n-1}P(k). \tag{3}$$

In addition, we notice that $P_p(k)$ has a new natural cutoff, $K_p$, which depends on $p$ and $N$ as follows (see Sec II.D in SM),

$$K_p \sim p^{n/(\gamma-1)}N^{1/(\gamma-1)}.$$

This helps us to evaluate the second moment of $P_p(k)$

$$\langle k_p^2 \rangle \sim \int_m^{K_p} k^2 p^{n-1} A k^{-\gamma} \sim p^{n-1}{K_p}^{3-\gamma} \sim p^{n-1+n\beta}N^{\beta},$$

where $\beta = (3-\gamma)/(\gamma-1)$.

Considering this, and plugging Eq. (3) into Eq. (14), and keeping the leading terms in the limit of large $N$, we obtain (see Sec II.D in SM for more details)

$$p_c \sim C(n)N^{-\delta/n} \sim C(n)\exp\left[-\delta\frac{\log N}{n}\right], \tag{4}$$

ORIGINAL UNEDITED MANUSCRIPT

where

$$\delta = \frac{\beta}{1+\beta} = \frac{3-\gamma}{2}.$$

and

$$C(n) = \phi^{1/n}, \qquad \phi = \left(\frac{3-\gamma}{\gamma-2}\frac{1}{m}\right)^{(\gamma-1)/2}.$$

From Eq. (4), it is easy to see that if $n \ll \log N$ then $p_c \to 0$, while if $n \sim \log N$ then $p_c$ is non-zero. The pre-factor $C(n)$ depends on $n$ but not in $N$.

Fig. 4(a) shows $p_c$ versus $\gamma$. It is known that for $2 < \gamma < 3$ and $n = 1$, if $N \to \infty$ then $p_c \to 0$ [34]. Also for $n = 5$, we can see that system size matters and $p_c$ decreases as $N$ increases. Fig. 4(b) shows that the scaling with $n/\log N$ of Eq. (4) is valid. Furthermore, it is seen in Fig. 4(b) that when $n$ is small or $N$ is large, such that $n/\log N \ll 1$ (in Fig. 4 it is 0.07), $p_c$ approaches 0. Fig. 4(c) supports the exponential scaling of $p_c$ versus $n^{-1}\log N$ obtained analytically in Eq. (4).

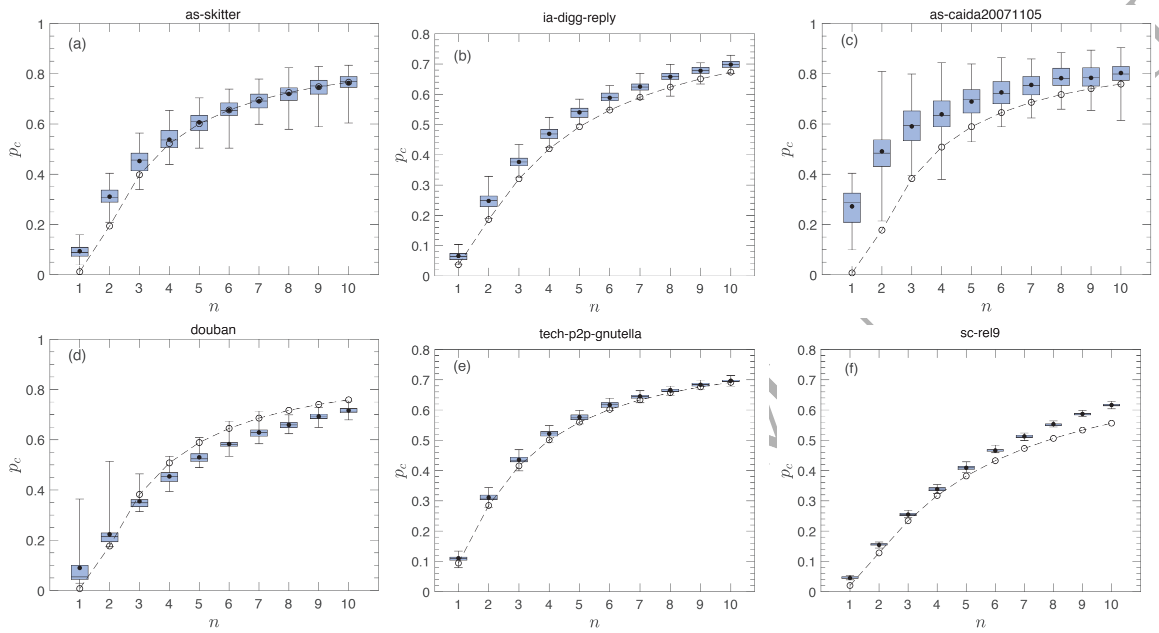

FIG. 5. Limited knowledge efficient immunization on six real word networks. Each panel is the result of 200 independent simulations. In each panel, black solid circles, horizontal lines and dash lines are mean, median value and analytical results of Eq. (14) respectively. In the simulations, we use the peak of second giant component to identify the critical point, $p_c$.

## Real Networks

Our analytical framework is derived and well supported, as shown above, on an ensemble of networks generated by random configuration model. Here we also test our analytical framework on six real-world data-set ranging from computer to social networks. They include (a) autonomous computer networks from Skitter project [42], (b) The reply network of the social news website Digg [43, 44], (c) network of autonomous systems of the Internet from the CAIDA project [42], (c) social friendship network from Douban [45], (e)tech-p2p, eDonkey peer to peer network [43, 46]. (f) sc-rel9, scientific computing network [43, 47]. Detailed information and statistical features of these networks are summarized in Sec IV of SM. To test our theory, we execute the limited knowledge immunization on the above real-world networks and use the peak of second giant component to determine the critical point $p_c$, see Fig. 5. Among the six real networks, Some are For specific $n$, the box diagram shows the distribution of critical point over 200 independent realizations, plotted together with the theoretical results. As seen in Fig. 5, we find that the critical thresholds $p_c$ for the empirical data are generally consistent with our theoretical predictions. Moreover, it is seen that our limited knowledge immunization approach is efficient to prevent outbreak even for small $n$ of order 10.

ORIGINAL UNEDITED MANUSCRIPT

## DISCUSSION

In summary, our results provide a framework for understanding and carrying out efficient immunization (or efficient attack) with limited knowledge. Especially in cases of global pandemics such as e.g., the current COVID-19, it is impossible to know the full interactions of all individuals and immunize the most centrals. We find that an effective way to limit spreading is obtaining information on a few ($n$ order of 10) individuals and targeting (testing or quarantine) the most central of these. For example, testers could stand at a supermarket and select a small number of people entering the store simultaneously. Information on the connections of these people e.g., the number of people they live or work closely with, where and how often they meet with other people etc. could be quickly obtained or small set through cell phone tracking and only the individual with the most connections in the group could be tested, quarantined or immunized. Our results demonstrate that even when this is done in small groups of people (low $n$ of order of 10), it is possible to obtain a significant decrease in global epidemics compared to randomly selecting individuals. In our model, this can be seen by the significantly reduced size of the giant component and the significantly high critical threshold $p_c$. Overall, these findings could help to develop efficient approaches for immunizing large networks and designing resilient infrastructure.

Finally, we wish to note the following two possible variations of our model. In our approach, once we get the degree of a node, we could also obtain partially information (some links) of neighboring nodes and utilized to target the highest degree node. We find, that adding this knowledge does not change the key results of the proposed model. For the detailed results, one can see in the Sec V of SM. Additionally, our immunization approach assumes the existence of a link or not as a binary process. Another variant of the model that would be interesting is to incorporate difference frequencies of interactions between different nodes. This would suggest that some nodes are likely to infect some of their neighbors more than others. Such a setting could be implemented by a weighted network, which would be a good topic for future study.

## METHODS

Suppose the degree distribution of a network is given by $P(k)$ and $F(k) = \sum_{s=0}^{k} P(s)$ being the cumulative probability that the degree of a randomly chosen node is less than or equal to $k$. Furthermore, at an arbitrary time $t$ during the iterative percolation process, assume the distribution of the original degree (including the immunized neighbors) of the remaining nodes is $P(k,t)$. Then, the degree distribution of the node which is immunized at time $t$ is given by

$$P_r(k,t) = F(k,t)^n - F(k-1,t)^n \equiv \Delta\left[F(k,t)^n\right], \tag{5}$$

where $F(k,t)$ is the cumulative distribution of $P(k,t)$. This formula can be recognized as being derived from *order statistics* giving the *maximum* of several independent random variables [48] (See Sec II.A in SM). For $k = 0$, Eq. (5) becomes $P_r(0,t) = F(0,t)^n$. Hence we define $F(k-1,t) = 0$, and then Eq. (5) is valid for $k \geq 0$.

In a limited knowledge immunization, each node's immunization changes the degree distribution of the remaining nodes in the following way

$$N(k,t+1) = N(k,t) - P_r(k,t), \tag{6}$$

where $N(k,t)$ is the number of nodes with degree $k$ at time $t$, and $P_r(k,t)$ is the likelihood that a node immunized at time $t$ has a degree $k$.

Then, plugging Eq. (5) into Eq. (6) gives,

$$N(k,t+1) = N(k,t) - \Delta\left[F(k,t)^n\right],$$

which becomes in the continuous limit,

$$\frac{\partial N(k,t)}{\partial t} = -\Delta\left[F(k,t)^n\right].$$

Substituting $N(k,t) = (N-t)P(k,t)$, we get

$$-P(k,t) + (N-t)\frac{\partial P(k,t)}{\partial t} = -\Delta\left[F(k,t)^n\right],$$

ORIGINAL UNEDITED MANUSCRIPT

and using $P(k,t) = \Delta F(k,t)$, we obtain,

$$\Delta\left[-F(k,t) + (N-t)\frac{\partial F(k,t)}{\partial t} + F(k,t)^n\right] = 0.$$

Note that $F(k=-1,t) = 0$, and thus the entire term inside the $\Delta$ is 0 for $k=-1$. Similarly, this implies that for $k=0$ and likewise for any $k \geq 0$ this term is also 0. Thus, we get the following simple ordinary differential equation,

$$(N-t)\frac{\partial}{\partial t}F(k,t) = F(k,t) - F(k,t)^n, \tag{7}$$

with the initial condition $F(k,t=0) = F(k)$. It can be shown (see Sec II.B in SM) that the solution of this ODE, Eq. (7) is

$$F(k,t) = \left(1 + \left(F(k)^{1-n} - 1\right)e^{(n-1)\log[(N-t)/N]}\right)^{-\frac{1}{n-1}}, \tag{8}$$

or equivalently,

$$F_p(k) = \left(1 + \left(F(k)^{1-n} - 1\right)p^{n-1}\right)^{-\frac{1}{n-1}}, \tag{9}$$

where $F_p(k)$ is the cumulative distribution of the degree after immunizing $1-p$ fraction of nodes. For $n=1$, the solution of Eq. (7) is $F_p(k) = F(k)$ as expected. Also Eq. (9) converges to $F(k)$ in the limit $n \to 1$.

We can now obtain the degree distribution of the occupied nodes after fraction $1-p$ nodes are immunized, which is given by

$$P_p(k) = \Delta F_p(k) = F_p(k) - F_p(k-1). \tag{10}$$

The equation for $v$, the probability of a randomly chosen link to lead to a node not in the giant component, is

$$1 - v = \sum_{k=0}^{\infty}\frac{kP(k)}{\langle k\rangle}P(\Theta|k)(1 - v^{k-1}), \tag{11}$$

where $P(\Theta|k)$ is the probability of a node to be occupied given its degree is $k$. This self-consistent equation can be recognized as being derived based on generating function method [3]. Using Bayes Theorem, we note that $P(k)P(\Theta|k) = P(\Theta)P(k|\Theta) = pP_p(k)$. Hence Eq. 11 becomes

$$1 - v = \frac{p}{\langle k\rangle}\sum_{k=0}^{\infty}kP_p(k)(1 - v^{k-1}). \tag{12}$$

The giant component $P_\infty$ can be found by

$$P_\infty = \sum_{k=0}^{\infty}P(k)P(\Theta|k)(1 - v^k) = p\sum_{k=0}^{\infty}P_p(k)(1 - v^k), \tag{13}$$

where $v$ is found from Eq. (12).

At criticality, we take the derivative of both sides of Eq. (12) and substitute $v=1$ representing the location where the first solution with $v<1$ exists, as opposed to only the $v=1$ solution. Thus, the critical condition is

$$1 = \frac{p_c}{\langle k\rangle}\sum_{k=0}^{\infty}k(k-1)P_{p_c}(k). \tag{14}$$

## SUPPLEMENTARY DATA

Supplementary data are available at NSR online.

ORIGINAL UNEDITED MANUSCRIPT

## FUNDING

G. D. thank the National Natural Science Foundation (61973143, 71974080 and 71690242), Y. L. thank the National Key R&D Program of China(2017YFC1200301), S.H. thank the Italian Ministry of Foreign Affairs and International Cooperation jointly with the Israeli Ministry of Science, Technology, and Space (MOST); the Israel Science Foundation, ONR, the Japan Science Foundation with MOST, BSF-NSF, ARO, the BIU Center for Research in Applied Cryptography and Cyber Security, and DTRA (HDTRA1-14-1-0017 and HDTRA-1-19-1-0016) for financial support.

## AUTHOR CONTRIBUTIONS

YL, GD, LS and SH designed the research, YL, HS and SB analyzed the model, YL, HS, GD and FW performed the experiments, all authors wrote the manuscript.

**Conflict of interest statement.** None declared.

ORIGINAL UNEDITED MANUSCRIPT

[1] Newman MEJ. The structure and function of complex networks. *SIAM Rev* 2003; **45**: 167–256.
[2] Albert R and Barabási AL. Statistical mechanics of complex networks. *Rev. Mod. Phys.* 2002; **74**: 47.
[3] Cohen R and Havlin S. Complex networks: structure, robustness and function. *Cambridge University Press*,2010.
[4] Cohen R, Havlin S, and Ben-Avraham D. Efficient immunization strategies for computer networks and populations. *Phys Rev Lett* 2003; **91**: 247901.
[5] Brockmann D and Helbing D. The hidden geometry of complex, network-driven contagion phenomena. *Science* 2013; **342**: 1337–1342.
[6] Shi D, Lü L and Chen G *et al.* Totally homogeneous networks. *Natl Sci Rev* 2019; **6**: 962–969.
[7] Liu YY, Csóka E and Zhou HJ *et al.* Core percolation on complex networks. *Phys Rev Lett* 2012; **109**: 205703.
[8] Liu YY, Slotine JJ, and Barabási AL. Controllability of complex networks. *Nature*; **473**: 167–173.
[9] Zhang Z and Zhang S. Towards understanding residual and dilated dense neural networks via convolutional sparse coding. *Natl Sci Rev* 2020; https://doi.org/10.1093/nsr/nwaa159.
[10] Watts DJ. A simple model of global cascades on random networks. *Proc Natl Acad Sci USA* 2002; **99**: 5766–5771.
[11] Tang D, Du W and Shekhtman LM *et al.* Predictability of real temporal networks. *Natl Sci Rev* 2020; **7**: 929–937.
[12] Albert R, Jeong H, and Barabási AL. Internet: Diameter of the world-wide web. *Nature* 1999; **401**: 130–131.
[13] Toroczkai Z and Bassler KE. Network dynamics: Jamming is limited in scale-free systems. *Nature* 2004; **428**: 716.
[14] Pastor-Satorras R and Vespignani A. Epidemic spreading in scale-free networks. *Phys Rev Lett* 2001; **86**: 3200–3.
[15] Watts DJ and Strogatz SH. Collective dynamics of ”small-world” networks. *Nature* 1998; **393**: 440–442.
[16] Gallos LK, Cohen R and Argyrakis P *et al.* Stability and topology of scale-free networks under attack and defense strategies. *Phys Rev Lett* 2005; **94**: 188701.
[17] Huang X, Gao J and Buldyrev SV *et al.* Robustness of interdependent networks under targeted attack. *Phys Rev E* 2011; **83**: 065101.
[18] Neumayer S, Zussman G and Cohen R *et al.* Assessing the vulnerability of the fiber infrastructure to disasters. *IEEE/ACM TON* 2011; **19**: 1610–1623.
[19] Helbing D. Globally networked risks and how to respond. *Nature* 2013; **497**: 51-59.
[20] Eubank S, Guclu H and Kumar VSA *et al.* Modelling disease outbreaks in realistic urban social networks. *Nature* 2004, **429**: 180–184.
[21] Stauffer D and Aharony A. *Introduction to percolation theory: revised second edition.* CRC press, 2014.
[22] Stanley HE. *Phase transitions and critical phenomena.* Clarendon Press, Oxford, 1971.
[23] Buldyrev SV, Parshani R and Paul G *et al.* Catastrophic cascade of failures in interdependent networks. *Nature* 2010; **464**: 1025–1028.
[24] Newman MEJ, Strogatz SH, and Watts DJ. Random graphs with arbitrary degree distributions and their applications. *Phys Rev E* 2001; **64**: 026118.
[25] Gao J, Buldyrev SV and Stanley HE *et al.* Networks formed from interdependent networks. *Nat Phys* 2012; **8**: 40–48.
[26] Dong G, Gao J and Du R *et al.* Robustness of network of networks under targeted attack. *Phys Rev E* 2013; **87**: 052804.
[27] Shekhtman LM, Danziger MM and Havlin S. Recent advances on failure and recovery in networks of networks. *Chaos Solitons Fractals* 2016; **90**: 28–36.
[28] Bunde A and Havlin S. Fractals and disordered systems. *Springer Science & Business Media*, 2012.
[29] Dong G, Fan J and Shekhtman LM *et al.* Resilience of networks with community structure behaves as if under an external field. *Proc Natl Acad Sci USA* 2018; **115**:6911–6915.
[30] Coniglio A, Nappi CR and Peruggi F *et al.* Percolation points and critical point in the ising model. *J Phys A-Math Gen* 1977; **10**: 205.

[31] Morone F and Makse HA. Influence maximization in complex networks through optimal percolation. *Nature* 2015; **524**: 65–68.
[32] Li M, Lü L and Deng Y *et al.* History-dependent percolation on multiplex networks. *Natl Sci Rev* 2020; **7**: 1296–1305.
[33] Albert R, Jeong H and Barabási AL. Error and attack tolerance of complex networks. *Nature* 2000; **406**: 378–82.
[34] Cohen R, Erez K and Ben-Avraham D *et al.* Resilience of the internet to random breakdowns. *Phys Rev Lett* 2000; **85**: 4626–8.
[35] Callaway DS, Newman MEJ and Strogatz SH *et al.* Network robustness and fragility: Percolation on random graphs. *Phys Rev Lett* 2000; **85**: 5468–71.
[36] Cohen R, Erez K and Ben-Avraham D *et al.* Breakdown of the internet under intentional attack. *Phys Rev Lett* 2001; **86**: 3682–5.
[37] Yan G, Tsekenis G and Barzel B *et al.* Spectrum of controlling and observing complex networks. *Nat Phys* 2015; **11**: 779–786.
[38] Yang Y, Wang J and Motter AE. Network observability transitions. *Phys Rev Lett* 2012; **109**: 258701.
[39] Liu YY, Slotine JJ and Barabási AL. Observability of complex systems. *Proc Natl Acad Sci USA* 2013; **110**: 2460–2465.
[40] Wu J, Deng HZ and Tan YJ *et al.* Vulnerability of complex networks under intentional attack with incomplete information. *J Phys A-Math Theor* 2007; **40**: 2665.
[41] Wu J, Tan YJ and Deng HZ *et al.* A Robustness Model of Complex Networks with Tunable Attack Information Parameter. *Chinese Phys Lett* 2007; **24**: 2138-2141.
[42] Leskovec J, Kleinberg J and Faloutsos C. Graph evolution: Densification and shrinking diameters. *ACM TKDD* 2007; **1**: 2.
[43] Rossi RA and Ahmed NK. The network data repository with interactive graph analytics and visualization. In *AAAI*, 2015.
[44] Choudhury MD, Sundaram H and John A *et al.* Social synchrony: Predicting mimicry of user actions in online social media. In *ICCSE* 2009; **4**: 151–158.
[45] Zafarani R and Liu H. Social computing data repository at asu, 2009.
[46] Opsahl T and Panzarasa P. Clustering in weighted networks. *Social networks* 2009; **31**: 155–163.
[47] Bader DA, Meyerhenke H and Sanders P *et al.* Graph partitioning and graph clustering. In *10th DIMACS Implementation Challenge Workshop*, 2012.
[48] Hoel PG. Introduction to mathematical statistics. *Inc. New York*, (2nd Ed), 1954.

ORIGINAL UNEDITED MANUSCRIPT
